# Identification of candidate UDP-glycosyltransferases involved in protopanaxadiol-type ginsenoside biosynthesis in *Panax ginseng*

**DOI:** 10.1038/s41598-018-30262-7

**Published:** 2018-08-06

**Authors:** Kyo Bin Kang, Murukarthick Jayakodi, Yun Sun Lee, Van Binh Nguyen, Hyun-Seung Park, Hyun Jo Koo, Ik Young Choi, Dae Hyun Kim, You Jin Chung, Byeol Ryu, Dong Young Lee, Sang Hyun Sung, Tae-Jin Yang

**Affiliations:** 1 College of Pharmacy and Research Institute of Pharmaceutical Sciences, Seoul National University, Seoul, 08826 Republic of Korea; 2 Department of Plant Science, Plant Genomics and Breeding Institute, Research Institute of Agriculture and Life Sciences, College of Agriculture and Life Sciences, Seoul National University, Seoul, 08826 Republic of Korea; 3 Department of Agriculture and Life Industry, Kangwon National University, Gangwon-do, 24341 Republic of Korea

## Abstract

Ginsenosides are dammarane-type or triterpenoidal saponins that contribute to the various pharmacological activities of the medicinal herb *Panax ginseng*. The putative biosynthetic pathway for ginsenoside biosynthesis is known in *P*. *ginseng*, as are some of the transcripts and enzyme-encoding genes. However, few genes related to the UDP-glycosyltransferases (UGTs), enzymes that mediate glycosylation processes in final saponin biosynthesis, have been identified. Here, we generated three replicated Illumina RNA-Seq datasets from the adventitious roots of *P*. *ginseng* cultivar Cheongsun (CS) after 0, 12, 24, and 48 h of treatment with methyl jasmonate (MeJA). Using the same CS cultivar, metabolomic data were also generated at 0 h and every 12–24 h thereafter until 120 h of MeJA treatment. Differential gene expression, phylogenetic analysis, and metabolic profiling were used to identify candidate UGTs. Eleven candidate UGTs likely to be involved in ginsenoside glycosylation were identified. Eight of these were considered novel UGTs, newly identified in this study, and three were matched to previously characterized UGTs in *P*. *ginseng*. Phylogenetic analysis further asserted their association with ginsenoside biosynthesis. Additionally, metabolomic analysis revealed that the newly identified UGTs might be involved in the elongation of glycosyl chains of ginsenosides, especially of protopanaxadiol (PPD)-type ginsenosides.

## Introduction

Korean ginseng (*Panax ginseng* Meyer, Araliaceae) is a traditional medicinal herb that has been widely used in East Asia for thousands of years^1^. Roots of *P*. *ginseng* and their extracts have been reported to exhibit many therapeutic properties, e.g., maintaining immune homeostasis, improving brain function, preventing cancer, and adjusting blood pressure^2–4^. Some of the pharmacological activities of ginseng have been attributed to *Panax*-specific ginsenosides, major triterpenoidal saponin constituents of ginseng^5^. Ginsenosides are classified into three subgroups based on their aglycone backbone structures: dammaranes, oleananes, and ocotillol triterpenes. *P*. *ginseng* ginsenosides are mainly of the dammarane-type, which are further classified as protopanaxadiol (PPD; ginsenoside Rb_1_, Rc, Rd, Rg_3_, Rh_2_, etc.) or protopanaxatriol (PPT; Re, Rg_1_, Rg_2_, Rh_1_, etc.), according to the number of hydroxyl groups^6^.

We previously revealed the putative biosynthetic pathway for dammarane-type ginsenosides^7,8^. Briefly, dammarenediol II is generated from isopentenyl diphosphate (IPP) and dimethylallyl diphosphate (DMAPP) by condensation and cyclization reactions, and these are successively catalysed by farnesyl diphosphate synthase (FPPS), squalene synthase (SQS), squalene epoxidase (SQE), and dammarenediol II synthase (DDS). The aglycone ginsenoside metabolites PPD and PPT, are biosynthesized from dammarenediol II through hydroxylation by protopanaxadiol synthase (PgPPDS, CYP716A47) and protopanaxatriol synthase (PgPPTS, CYP716A53v2). Glycosylation of PPD and PPT produces a variety of ginsenosides^9^. The glycosylation of triterpenoidal aglycones is catalysed by UDP-glycosyltransferases (UGTs) that use uridine diphosphate (UDP)-activated sugar molecules as sugar donors^10^. More than 150 ginsenosides have been identified from *Panax* spp.^6^. This diversity is attributed to many unique UGT-encoding genes^11–14^. However, only a few UGTs have been characterized for their substrate specificity: PgUGT71A27 (UGTPg1; PPD to compound K)^15^, PgUGT74AE2 (PPD to ginsenoside Rh_2_ and compound K to ginsenoside F_2_, respectively), PgUGT94Q2 (ginsenoside Rh_2_ to Rg_3_, F_2_ to Rd, respectively)^16^, UGTPg101 (PPT to ginsenoside F_1_, and further to Rg_1_), and UGTPg100 (PPT to ginsenoside Rh_1_, and F_1_ to Rg_1_)^10^. Many other UGTs associated with ginsenoside biosynthesis remain unidentified.

Methyl jasmonate (MeJA) is an important cellular regulator in plants, which is involved in seed germination, root growth, fertility, fruit ripening, senescence, and plant defence mechanisms^17^. MeJA is an effective elicitor of ginsenoside biosynthesis in cultured cells and adventitious roots of *P*. *ginseng*^18,19^. In our previous study, we applied integrated transcriptomics and metabolomics to investigate metabolism in MeJA-treated adventitious roots of various *P*. *ginseng* cultivars^7^. Increased ginsenoside content corresponded with expression levels of candidate genes in the downstream ginsenoside biosynthetic pathway, including *SQE* and *DDS*^7^; however, the dynamic regulatory mechanism for the final accumulation of ginsenoside, catalysed by UGTs, remains unknown.

Our previous study revealed that, of three ginseng cultivars, adventitious roots of the CS cultivar produced the lowest level of ginsenosides and related gene expression. However, treating CS with MeJA dramatically increased the ginsenoside content and corresponding transcription levels of candidate genes^7^. Therefore, we deduced that CS would be an appropriate cultivar in which to identify putative UGTs associated with ginsenoside biosynthesis in response to MeJA treatment. In this study, we used complementary transcriptomic and metabolomic approaches to investigate the UGT-catalysed biosynthesis of ginsenosides. Expression levels of UGT-related genes, and ginsenoside contents, were analysed in a time-dependent manner using MeJA-treated adventitious roots of *P*. *ginseng*, cultivar CS.

## Results and Discussion

### Identification of candidate UGTs in P. ginseng

**Figure 1:**
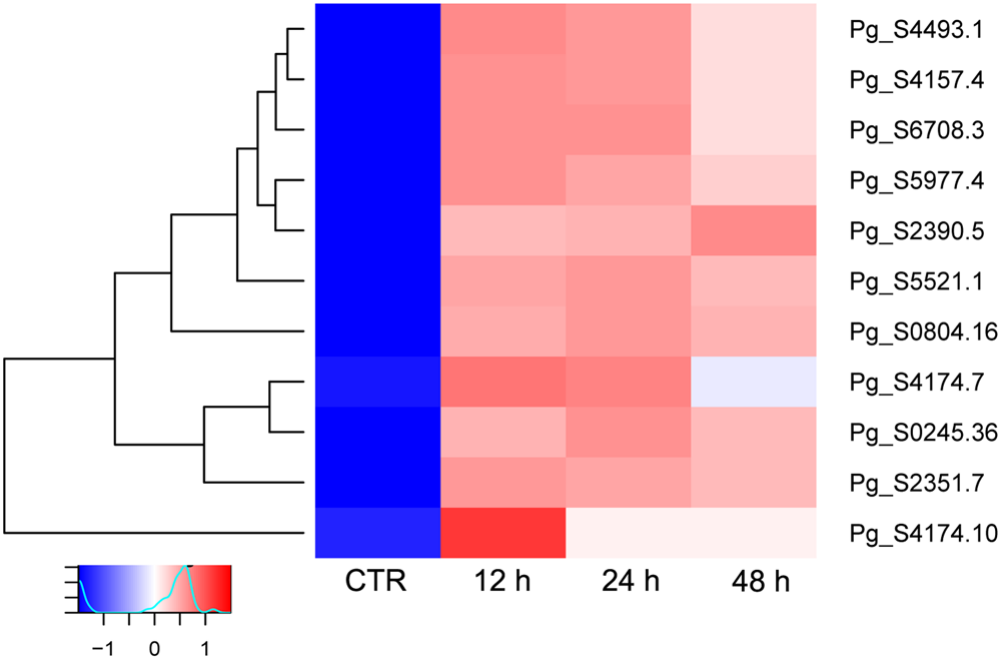
Differentially expressed *UDP-glycosyltransferase* (*UGT*) genes in *P*. *ginseng*. Using the edgeR Bioconductor package based on normalized fragments per kilobase per million (FPKM) read values of three biological replicates of each adventitious root sample, 11 UGT genes were identified as being differentially expressed between control (CTR: 0 h) and methyl jasmonate (MeJA)-treated samples (12, 24, and 48 h MeJA treatment). The heatmap showing hierarchical clustering FPKM was generated using the heatmap.2 function provided by the R-package gplots.

Using the most recent version of the *P*. *ginseng* annotation available in the Ginseng Genome Database^20^ (http://ginsengdb.snu.ac.kr/), 226 *UGT* genes were identified. Differential gene expression (DEG) analysis revealed that 11 of these were up-regulated after treatment with MeJA. Furthermore, using Interpro PROSITE (ID: PS00375), all 11 of these *UGT* genes encoded plant secondary product glycosyltransferase (PSPG) motifs, which are believed to be involved in binding the UDP moiety to glycosylate plant secondary metabolites^21,22^. Three of the 11 *UGT* genes (Pg_S4493.1, Pg_S4157.4, and Pg_S4174.7) matched with the previously characterized *P*. *ginseng* UGT-encoding genes KF377585.1 (*PgUGT1*), KM491309.1 (*PgUGT71A27*), and JX898529.1 (*PgUGT74AE2*) (Fig. 1). The remaining eight were considered novel UGT-encoding candidates.

**Figure 2:**
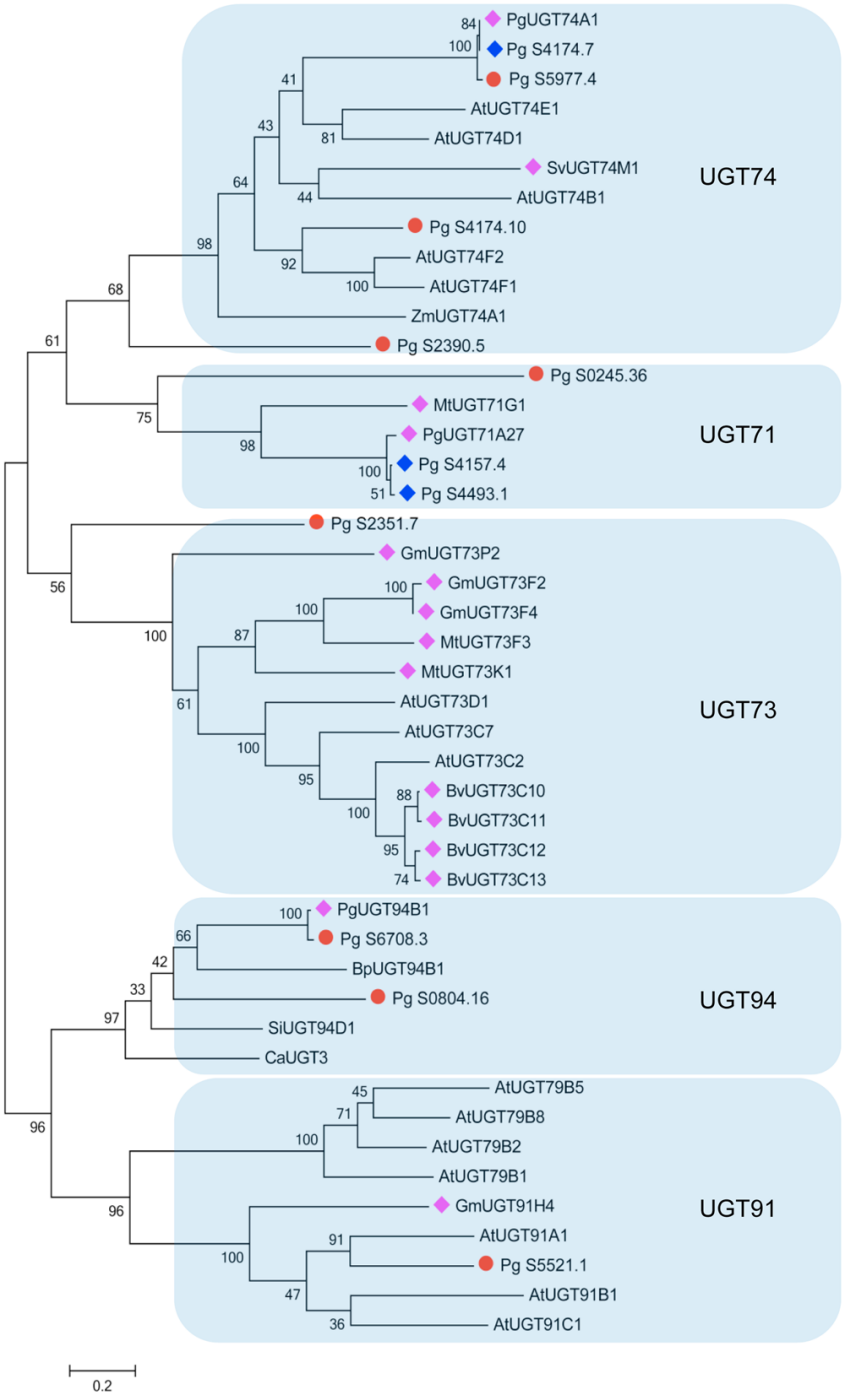
Phylogenetic analysis between candidate UGTs in *P*. *ginseng* and other plant UGTs including SiUGT94D1 (*Sesamum indicum*); CaUGT3 (*Catharanthus roseus*); AtUGT91A1, AtUGT91B1, AtUGT91C1, AtUGT79B1, AtUGT79B5, AtUGT79B2, AtUGT79B8, AtUGT73D1, AtUGT73C7, AtUGT73C2, AtUGT74F2, AtUGT74F1, AtUGT74B1, AtUGT74E1, AtUGT74D1 (*A*. *thaliana*); GmUGT73P2, GmUGT91H4, GmUGT73F2, GmUGT73F4 (*Glycine max*); MtUGT73F3, MtUGT73K1, MtUGT71G1 (*Medicago truncatula*); BvUGT73C10, BvUGT73C11, BvUGT73C12, BvUGT73C13 (*Barbarea vulgaris*); PgUGT71A27, PgUGT74A1 (*P*. *ginseng*); SvUGT74M1 (*Saponaria vacari*a); ZmUGT74A1 (*Zea mays*); BpUGT94B1 (*Bellis perennis*). The red circle indicates newly identified UGTs in this study and blue boxes represent previously identified UGTs of *P*. *ginseng*. Pink boxes show the UGTs involved in the triterpene pathway in other plant species. The transparent blue big rectangular boxes group these UGTs into different sub families including UGT74, UGT71, UGT73, UGT94 and UGT91.

Phylogenetic analysis was then performed with the candidate UGTs and known UGT proteins from other plant species (Fig. 2). Intriguingly, the candidate UGTs could be placed into the same groups as previously characterized UGT proteins from other plant species. UGTs belonging to the UGT74 family in *Arabidopsis thaliana*^23^ and *P*. *ginseng*^24^ recognize substrates of glucosinolates and ginsenoside Rh_2_, respectively. Evidence that members of the UGT74 family are involved in terpenoid biosynthesis has been reported in *A*. *thaliana*^25^ and *P*. *ginseng* (PgUGT71A27)^15^. We identified three new *UGT* genes (Pg_S4174.7, Pg_S4174.10 and Pg_S2390.5) belonging to the UGT74 family, along with a previously known *P*. *ginseng UGT* (*PgUGT74A1*).

**Figure 3:**
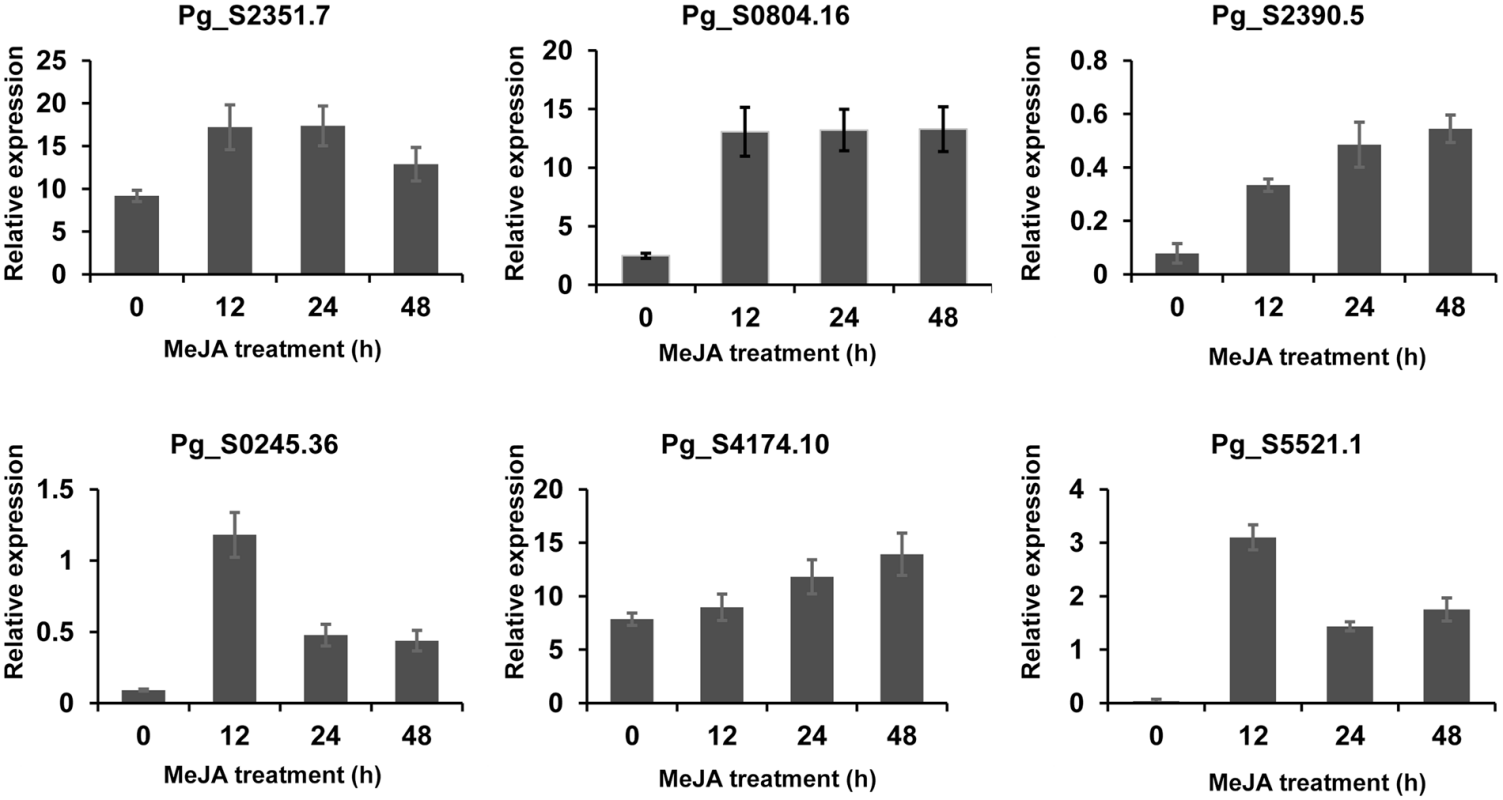
Relative expression *UDP-glycosyltransferase* genes differentially expressed between control (0 h) and methyl jasmonate-treated (after 12, 24 and 48 h) adventitious roots analysed by RT-quantitative polymerase chain reaction (RT-qPCR) assay. Results were normalized to a validated control gene, *PgActin*.

Members of the UGT73 family are involved in saponin glycosylation in Siberian ginseng (*Eleuthreococcus senticosus*, Araliaceae)^26^, and other plants such as *Barbarea vulgaris*, *Medicago truncatula*, and *Glycine max*^27^. Our analysis revealed that the UGT Pg_S2351.7 is related to the UGT73 family, suggesting a role for that UGT in glycosylation in *P*. *ginseng*. Similarly, the involvement of UGT94 and UGT91 family members in triterpene biosynthesis was previously found in *P*. *ginseng* (PgUGT94B1) and *G*. *max* (GmUGT91H4)^28^. Here, two novel *UGT* genes (Pg_S6708.3 and Pg_S0804.16) were grouped into the UGT94 family, and one (Pg_S5521.1) was grouped into the UGT91 family. To validate our RNA-Seq data, we randomly selected six genes for reverse-transcriptase quantitative PCR (qPCR) assays (Fig. 3). The resulting expression patterns were consistent with RNA-Seq data, except for one gene, Pg_S4174.10. We assume that this one discrepancy might have occurred because RNA-Seq read mapping is limited by the presence of highly identical paralog (>95% identity) copies of Pg_S4174.10 in *P*. *ginseng*.

### Ginsenoside profiles in Panax ginseng adventitious roots

**Figure 4:**
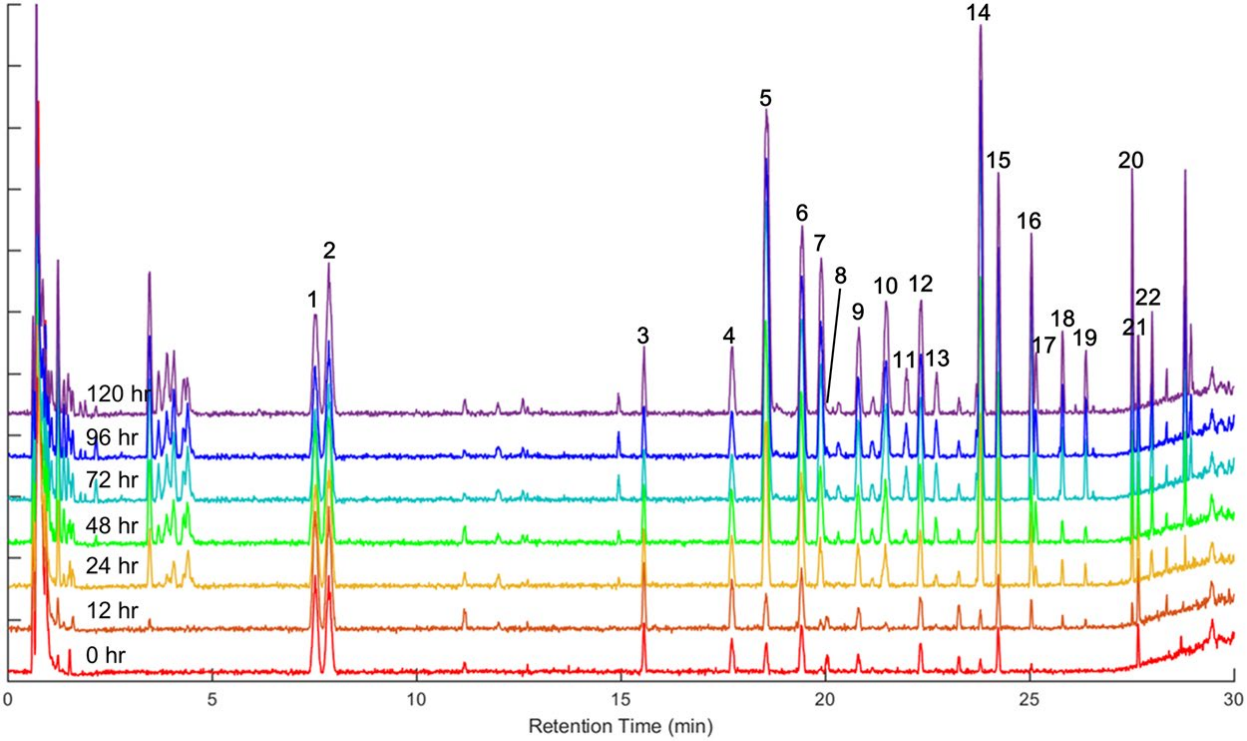
Liquid chromatography–mass spectrometry base peak ion chromatograms of adventitious roots of *P*. *ginseng* collected after 0–120 h treatment with methyl jasmonate. Numbers on chromatographic peaks annotate the ginsenosides identified and listed in Supplementary Table 1.

Adventitious root tissues from *P*. *ginseng* were prepared after 0, 12, 24, 48, 72, 96, and 120 h of MeJA treatment, and ginsenosides in extracts of those tissues were analysed using ultra-performance liquid chromatography–quad time of flight–mass spectrometry (UPLC-QTOF-MS) in triplicate (Fig. 4). Twenty-one major ginsenoside peaks were observed in LC-MS chromatograms. By comparing retention times and characteristic collision-induced dissociation (CID) fragment ions with reference compounds, 12 major ginsenosides (Rg_1_, Re, Rf, Rg_2_, Rb_1_, Rc, Ro, Rb_2_, Rb_3_, Rd, Rg_3_, and F_2_) were unambiguously identified. Other peaks were tentatively assigned by comparing their empirical molecular formulae, CID fragmentation patterns, and relative retention times, with those of previously published data (Supplementary Table 1). The analytical method used in this study was similar to that we used previously^7^, with some modifications to characterize a greater number of ginsenosides in a metabolomic profile. In particular, the peaks of two major ginsenosides, Re and Rg_1_, could be separated within a chromatogram.

**Figure 5:**
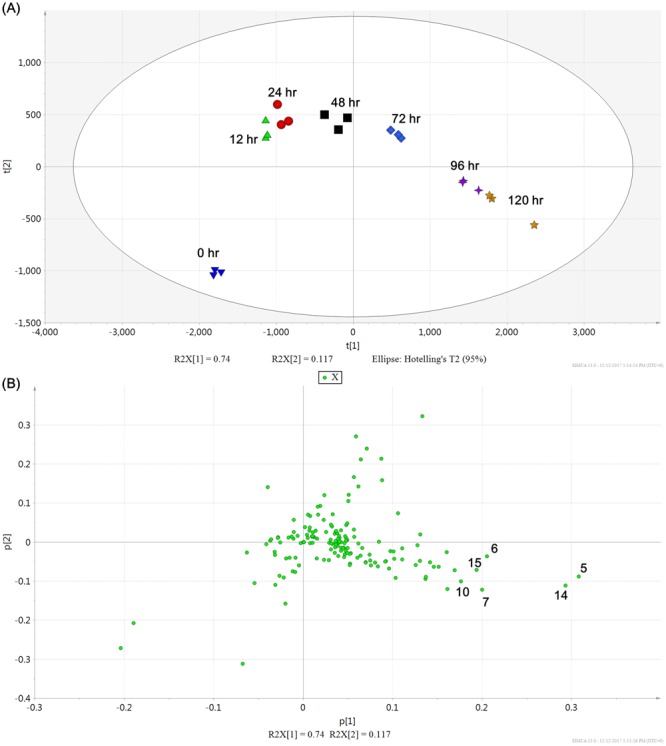
Principal component analysis (PCA) score plot (**A**) and loading plot (**B**) of liquid chromatography–mass spectrometry ginsenoside profiles of the adventitious roots if *P*. *ginseng* collected at different time points following treatment with methyl jasmonate. Numbers in the PCA loading plot annotate the compounds identified and listed in Supplementary Table 1.

Base peak ion (BPI) chromatograms showed that the levels of most ginsenosides were dramatically increased following MeJA treatment, but some – Rg_1_ (**1**), Re (**2**), and Ro (**8**) –showed relatively constant amounts. Intensities of chromatographic peaks at t_R_ 3–5 min were also significantly increased; however, these metabolites were not expected to be ginsenosides (expected molecular formula were C_18_H_30_O_8_ and C_19_H_32_O_10_) although they could not be identified precisely. For more detailed analysis of ginsenoside profiles, our LC-MS dataset was processed into a peak table including 156 ion markers using Mzmine 2 software^29^. Principle component analysis (PCA) was performed on the processed ginsenoside profile to visualize time-dependent metabolic differences. In the PCA score plot, it is easy to recognize the ginsenoside accumulation pattern by increasing principle component 1 (PC1) (Fig. 5A). Between 0 and 120 h of MeJA elicitation, the PC1 value showed a constant increase and accounted for 75.2% of the total variance in the dataset. The PCA loading plot indicates that most ginsenosides correlate positively with PC1, implying that the levels of almost every ginsenoside increased after MeJA elicitation. The ginsenosides Rb_1_ (**5**), Rc (**7**), Rb_2_ (**10**), and Rd (**14**), and malonyl ginsenosides Rb_1_ (**6**) and Rd (**15**) showed especially high positive correlations with PC1, suggesting that biosynthesis of these ginsenosides is significantly induced by MeJA (Fig. 5B).

**Figure 6:**
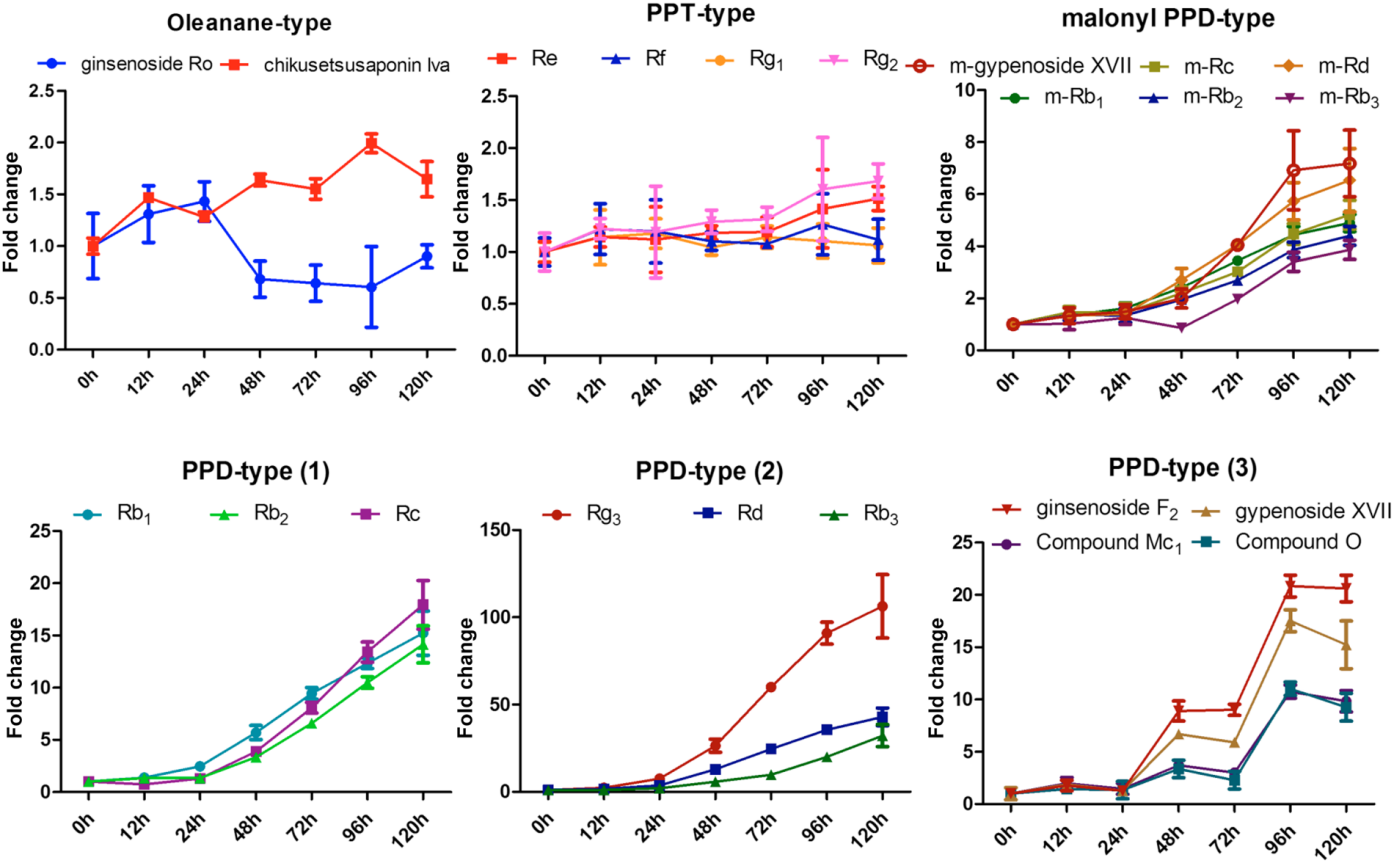
Relative abundances (fold changes) of the major groups of ginsenosides in adventitious roots of *Panax ginseng* treated with methyl jasmonate and analysed by liquid chromatography–mass spectrometry. Error bars represent standard deviations from the mean of three replicates. PPD, protopanaxadiol; PPT, protopanaxatriol.

Time-dependent accumulation patterns of the major ginsenosides were compared based on the ion intensity of their LC-MS profiles. Fold-changed relative abundance according to time after MeJA treatment was calculated using the Mzmine peak ion table (Fig. 6). As shown in Fig. 6, and as also found by Oh *et al*., the amounts of PPT-type and oleanane-type ginsenosides were not affected by MeJA treatment^30^, while the levels of PPD-type ginsenosides significantly increased with MeJA treatment and most PPD-type ginsenosides started to accumulate after 48 h. Our previous study suggested that PPD-type ginsenosides are involved in defence mechanisms against biotic stresses, while PPT-types are involved in mechanisms against abiotic stresses^30^. The ginsenosides Rg_3_, Rd, and Rb_3_ showed the most dramatic accumulation, with 106.3, 43.0, and 32.2-fold increases, respectively, after 120 h. Contents of the ginsenosides Rb_1_, Rb_2_, and Rc also significantly increased, with 15.2, 14.2, and 17.9-fold changes, respectively. The accumulation patterns of the ginsenoside F_2_, compounds Mc_1_ and O, and gypenoside XVII suggested that they became saturated after 96 h. Malonylated ginsenosides, a group of derivatives that are compartmentalized in the vacuole^31^, also increased in content, but these changes were relatively small compared with non-malonylated ginsenosides.

### A putative biosynthetic pathway and biological roles of ginsenosides

**Figure 7:**
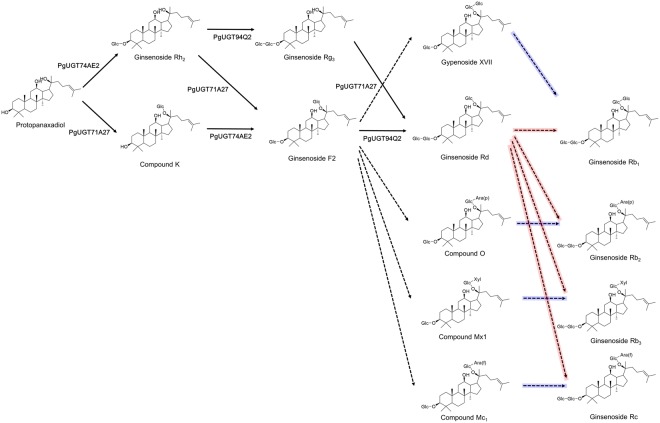
Proposed pathway of protopanaxadiol (PPD)-type ginsenoside biosynthesis in adventitious roots of *Panax ginseng*. Dotted arrows represent glycosylation steps for which the mediating UDP-glycosyltransferases (UGTs) are as yet unknown. Blue and red lines show two possible pathways for the addition of two glucose residues at the C3-OH position (see text). *Glc*, β-d-glucopyranosyl; *Ara*(*p*), α-l-arabinopyranosyl; *Ara*(*f*), α-l-arabinofuranosy; *Xyl*, β-d-xylopyranosyl.

Figure 7 shows the possible biosynthetic pathways of the PPD-type ginsenosides that significantly accumulated after MeJA treatment. These pathways are proposed based on their sugar moiety sequences. Of the compounds identified, several have two glucose residues at the C3-OH position, and there are two possible pathways for the biosynthesis of ginsenosides Rb_1_, Rb_2_, Rb_3_ and Rc (see red and blue lines in Fig. 7). The red pathway follows the route from ginsenoside Rg_3_ to ginsenosides Rb_1_, Rb_2_, Rb_3_, and Rc through ginsenoside Rd. Several different UGTs might be involved in this step by adding different glucose moieties, such as Glc, Xyl, Ara(*p*) and Ara(*f*). The blue pathway uses different substrates, but the reaction is the same: adding a glucose residue to 3-*O*-glucoside. In MeJA-treated adventitious roots of *P*. *ginseng*, the fold-changes of ginsenosides Rb_1_, Rb_2_, Rb_3_, and Rc show similar patterns as in Fig. 6, implying that these compounds are produced by a single UGT, or are co-controlled by very similar UGTs. Therefore, we suggest that the biosynthesis of ginsenosides Rb_1_, Rb_2_, Rb_3_, and Rc follows the blue pathway shown in Fig. 7.

Malonylation is a common form of plant metabolism used to store toxic materials separately in vacuoles^32^. The fact that ginsenosides undergo malonylation therefore provides evidence for the role of ginsenosides in defence. Malonylated ginsenosides significantly elicited by MeJA treatment in our study were Rb_1_, Rb_2_, Rb_3_, Rc, Rd, and gypenoside XVII. We therefore suggest that these ginsenosides are the metabolites related to plant defence in *P*. *ginseng*, and propose that the 11 UGTs – including eight novel UGTs – identified in this study may contribute to the glycosylation of these ginsenosides.

## Conclusion

We investigated time-dependent changes in UGT-related gene expression and ginsenoside content of MeJA-treated adventitious roots of the *P*. *ginseng* cultivar CS, which was previously shown to markedly increase ginsenoside biosynthesis upon treatment with MeJA. Transcriptomic analysis identified 11 candidate UGTs, including eight novel and three previously characterized UGTs. Metabolic profiling and phylogenetic analysis revealed that these candidate UGTs are strongly associated with ginsenoside biosynthesis. Furthermore, these newly identified UGTs might be related to steps involving the elongation of ginsenoside glycosyl chains, especially in PPD-type ginsenosides. Further functional characterization studies will elucidate the specific substrates of these UGTs in *P*. *ginseng*.

## Materials and Methods

### Plant materials

Adventitious roots of *P*. *ginseng* (CS cultivar) were cultured as previously described^33^ and amplified in bioreactors containing 1 L Schenk and Hildebrandt liquid medium supplemented with 3 mg/L indole-3-butyric acid and 5% sucrose. Three grams of these fresh proliferated roots was transferred into 250-mL flasks containing the same medium supplemented with 200 µM MeJA and cultured at 23 ± 1 °C on a rotary shaker at 100 rpm under constant dark conditions. We selected 21 flasks containing healthy growing adventitious roots and harvested these with three replications for each condition: 0, 12, 24, 48, 72, 96, and 120 h after initiation of MeJA treatment.

### Chemicals and reagents

Standard references of 12 ginsenosides were purchased from Chengdu Biopurify Phytochemicals Co., Ltd. (Chengdu, China; Rb_1_, Rb_2_, Rf, Rg_2_, Rg_3_, Ro, and F_2_) and Chromadex (Irvine, CA, USA; Rb_3_, Rc, Rd, and Re), or were provided by the Korean Food and Drug Administration (Rg_1_). Chemicals for tissue culture were purchased from Duchefa (Haarlem, The Netherlands), and high-performance liquid chromatography (HPLC) grade solvents were purchased from Avantor Performance Materials Inc. (Center Valley, PA, USA). All other chemicals were purchased from Sigma–Aldrich (St. Louis, MO, United States).

### RNA isolation and transcriptome sequencing using the Illumina platform

Total RNA was isolated from MeJA-treated adventitious roots after 12, 24 and 48 h using the RNeasy Plant Kit (QIAGEN, Germany) according to the manufacturer’s instructions. After examining its quality and quantity using a bioanalyzer (Agilent Technologies, USA), about 2 μg total RNA was used to independently construct RNA-Seq libraries with an insert size of 300 bp using the Illumina TruSeq RNA Sample Preparation Kit according to the manufacturer’s instructions. Pooled libraries were sequenced with a paired-end read length of 150 bp using the Illumina NextSeq 500 platform at LabGenomics Co. (Seongnam, Korea). Raw reads were deposited into the National Center for Biotechnology Information Sequencing Read Archive. Accession numbers are provided in Table 1. Prior to differential gene expression analysis, reads containing bacterial contaminants were removed by mapping against available bacterial genomes using BWA^34^. Adaptor contamination was then removed using the NGS QC Toolkit (v2.3.3)^35^.

**Table 1:** Summary of transcriptome data generated from adventitious roots of Cheongsun (CS) *Panax ginseng* cultivars.

CS adventitious roots	Raw data		Filtered data		SRA accession no.
	Total no. of reads	Length (bp)	Total no. of reads	Length (bp)	
CS, replicate 1*	16,731,664	2,321,621,040	15,291,398	2,115,447,628	SRR2132332
CS, replicate 2*	14,306,820	1,977,938,763	12,831,592	1,766,225,247	SRR1688723
CS, replicate 3*	14,801,822	2,056,937,756	13,407,302	1,857,154,376	SRR2132333
CS, 12 h replicate 1	13,820,382	1,893,236,025	12,221,130	1,665,343,695	SRR6384120
CS, 12 h replicate 2	13,549,482	1,889,923,163	11,912,380	1,655,061,269	SRR6384121
CS, 12 h replicate 3	14,884,004	1,951,092,722	12,996,188	1,689,391,983	SRR6384122
CS, 24 h replicate 1	13,708,904	1,903,958,122	12,125,782	1,677,279,963	SRR6384123
CS, 24 h replicate 2	14,492,432	2,015,422,717	12,732,674	1,762,648,809	SRR6384124
CS, 24 h replicate 3	13,891,838	1,927,722,530	12,173,452	1,681,509,097	SRR6384125
CS, 48 h replicate 1	11,055,530	1,531,553,431	9,669,218	1,333,220,591	SRR6384126
CS, 48 h replicate 2	14,083,014	1,945,787,757	12,425,406	1,709,114,504	SRR6384127
CS, 48 h replicate 3	15,591,720	2,160,145,356	13,525,934	1,864,372,366	SRR6384128
Total	170,917,612	23,575,339,382	151,312,456	20,776,769,528	

*Replicated transcriptomes of CS obtained from a previous report^38^.

### Identification of UGTs in P. ginseng and differential gene expression analysis

The current version of the ginseng gene set (IPGA_v1.1) was retrieved from the Ginseng Genome Database^20^ (https://ginsengdb.snu.ac.kr). Protein domains and motifs of UGT genes in *P*. *ginseng* were identified using InterProScan^36^. Trimmed, high-quality RNA-Seq reads were mapped to the ginseng gene set to calculate fragments per kilobase per million (FPKM) using RSEM^37^. The bioconductor package edgeR^38^ was used to identify differentially expressed genes in MeJA-treated samples. Genes exhibiting changes greater than two-fold with a significant false discovery rate (FDR) of 0.001 were considered differentially expressed.

### Quantitative RT-PCR assay

Total RNA was isolated from the adventitious roots of *P*. *ginseng* cultivar CS treated with MeJA using the RNeasy Plant Kit (QIAGEN, Germany) according to the manufacturer’s protocol. RNA quality and quantity was examined using formaldehyde agarose gel electrophoresis. cDNA was synthesized using 1 µg of RNA (Invitrogen, USA), and the cDNA was diluted to 1/10 for use in quantitative polymerase chain reaction (qPCR). qPCR was performed using SYBR Green Power PCR Master Mix (Applied Biosystems, Foster City, CA) in the Roche Lightcycler 480 Realtime PCR system (Roche, Indianapolis, IN). The thermal cycling conditions for PCR were: 95 °C for 5 min, 30 cycles at 95 °C for 5 s, 60 °C for 30 s and 72 °C for 30 s, followed by 72 °C for 5 min. qPCR was carried out in duplicate for each sample. The qPCR primers used in this study are listed in Supplementary Table 2.

### Ginsenoside analysis using LC–MS

Twenty milligrams of freeze-dried sample powder was sonicated with 1 mL of 70% ethanol for 90 min at room temperature. Extracts were centrifuged at 17,000 × *g* for 3 min, then 500 μL of each supernatant was dried using nitrogen gas and resuspended in 1 mL 50% methanol. Prepared samples were analyzed using a Waters ACQUITY ultra-performance liquid chromatography (UPLC) system (Waters Co., Milford, MA, USA) coupled with a Waters Xevo G2 quad time-of-flight (QTOF) mass spectrometer (Waters MS Technologies, Manchester, UK), which was equipped with an electrospray interface (ESI). Separation was performed on an ACQUITY UPLC BEH C_18_ (100 mm × 2.1 mm, 1.7 μm, Waters Co.) column. The mobile phase consisted of 0.1% formic acid in water (A) and acetonitrile (B) with the following gradient condition: 20% B (0–9 min); 20–30% B (9–14 min); 30% B (14–17 min); 30–32% B (17–21 min); 32–42.5% B (21–26 min); 42.5–90% B (26–30 min). The flow rate of the mobile phase was 0.3 mL/min, and the column temperature was maintained at 25 °C. Analyses of the samples (1.0 μL injected into the partial loop in needle overfill mode) were performed in negative ion mode in the 100–1500 Da range, with acquisition times of 0.2 s in centroid mode. ESI conditions were set as follows: capillary voltage 3.5 kV, con voltage 45 V, source temperature 120 °C, desolvation temperature 300 °C, cone gas flow 50 L/h, and desolvation gas flow 800 L/h. High-purity nitrogen was used as the nebulizer and auxiliary gas, and argon was used as the collision gas. The mass spectrometer was calibrated using sodium formate over a range of 100–1500 Da; leucine enkephalin (*m/z* 554.2615 [M –H ]^−^) was used as the lockmass to ensure mass accuracy and reproducibility. To identify ginsenosides, collision-induced dissociation (CID) data were recorded using MS^E^ methodology^39^. Low collision energy to detect precursor ions was set to 4 eV, and the high collision energy for fragmentation was set to 40–45 eV.

### LC–MS Data processing and multivariate analysis

Mass spectrometry (MS) ion markers were extracted from liquid chromatography–mass spectrometry (LC-MS) raw data using Mzmine2 2.30 software^40^. Mass ion detection was performed with the noise level set to 2000, and based on the detected mass list, peak lists were built with criteria as follows: minimum time span of 0.02 min, minimum height of 3000, and *m/z* tolerance of 0.006 Da (or 10.0 ppm). Chromatographic deconvolution was achieved using the baseline cut-off algorithm (minimum peak height of 3000, peak duration range of 0.02–0.30 min, and baseline level of 1000). Chromatograms were de-isotoped using the isotopic peaks grouper algorithm with an *m/z* tolerance of 0.006 Da (or 10.0 ppm) and a t_R_ tolerance of 0.1 min. Peak lists for samples were aligned together using the join aligner module (*m/z* tolerance at 0.006 Da or 20 ppm, absolute t_R_ tolerance at 0.3 min, weight for *m/z* of 70, and weight for t_R_ of 30), and the aligned peak list was eventually gap-filled with the peak finder module (intensity tolerance at 50%, *m/z* tolerance at 0.006 Da or 10.0 ppm, and absolute t_R_ tolerance of 0.2 min). Peaks from MS contaminants were identified by blank injection, and duplicate peaks were manually removed from the aligned and gap-filled peak list. Principal component analysis (PCA) was performed using SIMCA 13.0 software (Umetrics, Umeå, Sweden), with Pareto scaling of data.

## Electronic supplementary material

Supplementary Dataset 1

## References

[1] Dharmananda S (2002). The nature of ginseng: traditional use, modern research and the question of dosage. Herb. Gram.

[2] Vogler BK, Pittler MH, Ernst E (1999). The efficacy of ginseng. A systematic review of randomised clinical trials. Eur J Clin Pharmacol.

[3] Kang S, Min H (2012). Ginseng, the ‘Immunity Boost’: The Effects of Panax ginseng on Immune System. J Ginseng Res.

[4] Coleman CI, Hebert JH, Reddy P (2003). The effects of *Panax ginseng* on quality of life. J Clin Pharm Ther.

[5] Attele AS, Wu JA, Yuan CS (1999). Ginseng pharmacology: multiple constituents and multiple actions. Biochem Pharmacol.

[6] Christensen LP (2009). Ginsenosides chemistry, biosynthesis, analysis, and potential health effects. Adv Food Nutr Res.

[7] Lee, Y. S. *et al*. Integrated Transcriptomic and Metabolomic Analysis of Five *Panax ginseng* Cultivars Reveals the Dynamics of Ginsenoside Biosynthesis. *Front Plant Sci* **8** (2017). 10.3389/fpls.2017.01048 PMC5474932 28674547

[8] Kim, N. H. *et al*. Genome and evolution of the shade-requiring medicinal herb *Panax ginseng*. *Plant Biotechnol J*, 10.1111/pbi.12926 (2018). 10.1111/pbi.12926 PMC6181221 29604169

[9] Kim YJ, Lee OR, Oh JY, Jang MG, Yang DC (2014). Functional analysis of 3-hydroxy-3-methylglutaryl coenzyme a reductase encoding genes in triterpene saponin-producing ginseng. Plant Physiol.

[10] Wei W (2015). Characterization of *Panax ginseng* UDP-Glycosyltransferases Catalyzing Protopanaxatriol and Biosyntheses of Bioactive Ginsenosides F1 and Rh1 in Metabolically Engineered Yeasts. Mol Plant.

[11] Sun, C. *et al*. De novo sequencing and analysis of the American ginseng root transcriptome using a GS FLX Titanium platform to discover putative genes involved in ginsenoside biosynthesis. *BMC Genomics* **11** (2010). 10.1186/1471-2164-11-262 PMC2873478 20416102

[12] Chen S (2011). 454 EST analysis detects genes putatively involved in ginsenoside biosynthesis in *Panax ginseng*. Plant Cell Rep.

[13] Luo, H. M. *et al*. Analysis of the transcriptome of *Panax notoginseng* root uncovers putative triterpene saponin-biosynthetic genes and genetic markers. *BMC Genomics* **12** (2011). 10.1186/1471-2164-12-S5-S5 PMC3287501 22369100

[14] Li, C. F. *et al*. Transcriptome analysis reveals ginsenosides biosynthetic genes, microRNAs and simple sequence repeats in *Panax ginseng* C. A. Meyer. *BMC Genomics* **14** (2013). 10.1186/1471-2164-14-245 PMC3637502 23577925

[15] Yan X (2014). Production of bioactive ginsenoside compound K in metabolically engineered yeast. Cell Res.

[16] Jung SC (2014). Two Ginseng UDP-Glycosyltransferases Synthesize Ginsenoside Rg(3) and Rd. Plant Cell Physiol.

[17] Cheong JJ, Choi YD (2003). Methyl jasmonate as a vital substance in plants. Trends Genet.

[18] Kim YS, Hahn EJ, Murthy HN, Paek KY (2004). Adventitious root growth and ginsenoside accumulation in *Panax ginseng* cultures as affected by methyl jasmonate. Biotechnol Lett.

[19] Thanh NT, Murthy HN, Yu KW, Hahn EJ, Paek KY (2005). Methyl jasmonate elicitation enhanced synthesis of ginsenoside by cell suspension cultures of *Panax ginseng* in 5-I balloon type bubble bioreactors. Appl Microbiol Biot.

[20] Jayakodi, M. *et al*. Ginseng Genome Database: an open-access platform for genomics of *Panax ginseng*. *BMC Plant Biol* **18** (2018). 10.1186/s12870-018-1282-9 PMC5898050 29649979

[21] Gachon CMM, Langlois-Meurinne M, Saindrenan P (2005). Plant secondary metabolism glycosyltransferases: the emerging functional analysis. Trends Plant Sci.

[22] Vogt T, Jones P (2000). Glycosyltransferases in plant natural product synthesis: characterization of a supergene family. Trends Plant Sci.

[23] Grubb CD (2014). Comparative analysis of Arabidopsis UGT74 glucosyltransferases reveals a special role of UGT74C1 in glucosinolate biosynthesis. Plant J.

[24] Kim, S. C. *et al*. UDP-glycosyltransferase derived from ginseng and use thereof (2017).

[25] Caputi L, Malnoy M, Goremykin V, Nikiforova S, Martens S (2012). A genome-wide phylogenetic reconstruction of family 1 UDP-glycosyltransferases revealed the expansion of the family during the adaptation of plants to life on land. Plant J.

[26] Hwang, H. S., Lee, H. & Choi, Y. E. Transcriptomic analysis of Siberian ginseng (Eleutherococcus senticosus) to discover genes involved in saponin biosynthesis. *BMC Genomics* **16** (2015). 10.1186/s12864-015-1357-z PMC4369101 25888223

[27] Thimmappa R, Geisler K, Louveau T, O’Maille P, Osbourn A (2014). Triterpene Biosynthesis in Plants. Annual Review of Plant Biology, Vol 65.

[28] Shibuya M, Nishimura K, Yasuyama N, Ebizuka Y (2010). Identification and characterization of glycosyltransferases involved in the biosynthesis of soyasaponin I in *Glycine max*. FEBS Lett.

[29] Pluskal, T., Castillo, S., Villar-Briones, A. & Oresic, M. MZmine 2: Modular framework for processing, visualizing, and analyzing mass spectrometry-based molecular profile data. *BMC Bioinformatics* **11** (2010). 10.1186/1471-2105-11-395 PMC2918584 20650010

[30] Oh JY (2014). Investigation of ginsenosides in different tissues after elicitor treatment in *Panax ginseng*. J Ginseng Res.

[31] Kochkin DV (2013). Malonyl-ginsenoside content of a cell-suspension culture of *Panax japonicus* var. repens. Phytochemistry.

[32] Taguchi G (2010). Malonylation is a key reaction in the metabolism of xenobiotic phenolic glucosides in Arabidopsis and tobacco. Plant J.

[33] Jayakodi M (2014). Transcriptome profiling and comparative analysis of *Panax ginseng* adventitious roots. J Ginseng Res.

[34] Li H, Durbin R (2009). Fast and accurate short read alignment with Burrows-Wheeler transform. Bioinformatics.

[35] Patel, R. K. & Jain, M. NGS QC Toolkit: A Toolkit for Quality Control of Next Generation Sequencing Data. *Plos One* **7** (2012). 10.1371/journal.pone.0030619 PMC3270013 22312429

[36] Jones P (2014). InterProScan 5: genome-scale protein function classification. Bioinformatics.

[37] Li, B. & Dewey, C. N. RSEM: accurate transcript quantification from RNA-Seq data with or without a reference genome. *BMC Bioinformatics* **12** (2011). 10.1186/1471-2105-12-323 PMC3163565 21816040

[38] Robinson MD, McCarthy DJ, Smyth GK (2010). edgeR: a Bioconductor package for differential expression analysis of digital gene expression data. Bioinformatics.

[39] Plumb RS (2006). UPLC/MSE; a new approach for generating molecular fragment information for biomarker structure elucidation. Rapid Commun Mass Sp.

[40] Lee YS (2017). Comparative analysis of the transcriptomes and primary metabolite profiles of adventitious roots of five *Panax ginseng* cultivars. J Ginseng Res.

